# Foundations and Operational Mechanics of Value-Based Care

**DOI:** 10.7759/cureus.112956

**Published:** 2026-07-19

**Authors:** Mohammad Shibly Khan, Mamdouh Falih Althaqeel, Elmuez Eltayeb Elnaiem, Manal A Elimam, Omar A Alayed, Shibli Sayeed, Isameldin Abdelrahim, Talal Elfadil Mahdi, Safa M Alsaid, Mysoon Mohamed Elamin Saeed, Ahmed A Alfaify, Ahmed M Kheir

**Affiliations:** 1 Health Excellence, King Salman Hospital, Riyadh First Health Cluster, Riyadh, SAU; 2 Hospital Administration, King Salman Hospital, Riyadh First Health Cluster, Riyadh, SAU; 3 Quality and Patient Safety, King Salman Hospital, Riyadh First Health Cluster, Riyadh, SAU; 4 Health Program and Chronic Disease, Ministry of Health, Riyadh, SAU; 5 Family Medicine, Dr. Sulaiman Alhabib Medical Group, Riyadh, SAU; 6 Public Health, Ministry of Health, Riyadh, SAU; 7 Community Health Excellence, Riyadh First Health Cluster, Riyadh, SAU; 8 Population Health Management and Research, Riyadh First Health Cluster, Riyadh, SAU; 9 Infection Prevention and Control, King Salman Hospital, Riyadh, SAU; 10 Clinical Immunology, King Fahad Hospital, Al-Baha, SAU; 11 Quality and Patient Safety, Prince Sultan Military Medical City, Riyadh, SAU

**Keywords:** healthcare transformation, outcome-based finance, saudi arabia, value-based healthcare, vision 2030

## Abstract

The global escalation of healthcare expenditures alongside fluctuating clinical outcomes has forced a paradigm shift from volume-driven reimbursement frameworks to value-based healthcare (VBHC) models. This review article outlines the fundamental architectural principles, structural mechanisms, and financial configurations underpinning value-based delivery systems, with a particular focus on macro-level healthcare system transformation. VBHC fundamentally realigns institutional and clinical incentives by substituting the legacy fee-for-service methodology with outcome-linked reimbursement protocols. The structural operationalization of this model depends on four primary pillars: proactive preventive interventions, seamlessly integrated care coordination across multi-disciplinary provider networks, advanced data interoperability utilizing real-world evidence, and comprehensive financial risk-sharing agreements. Globally, this transition manifests through diverse regulatory initiatives, notably the Centers for Medicare & Medicaid Services strategies in the United States and the Health Sector Transformation Program under Saudi Vision 2030 in the Kingdom of Saudi Arabia. In the Saudi ecosystem, transformation is driven by the establishment of vertically integrated regional health clusters, strategic purchasing through the Center for National Health Insurance (CNHI), and private sector capitation rules enforced by the Council of Health Insurance (CHI). Despite complex execution bottlenecks - including systemic data fragmentation, provider cultural inertia, and the logistical challenges of micro-costing methodologies such as time-driven activity-based costing - the structural transition toward value-based frameworks represents an essential pathway for long-term fiscal sustainability, reduced clinical variation, and optimized population health outcomes. This article synthesizes contemporary literature to provide a systemic blueprint of the operational mechanics, economic incentives, and macro-level challenges shaping modern healthcare systems.

## Introduction and background

Imperative for healthcare transformation

For many years, international healthcare delivery networks operating under insurance-driven and multi-payer frameworks have struggled with an unsustainable path of rapidly rising operational costs alongside inconsistent clinical results [1]. Legacy healthcare architectures, particularly those built on fee-for-service models, were largely designed to address acute, episodic medical events rather than the continuous, multi-faceted complexities of chronic diseases and aging populations [1,2]. It has been widely recognized by healthcare policymakers, academic institutional leaders, and clinical directors that the structural root of the fiscal crisis lies within the underlying payment mechanisms that fund medical delivery [2,3]. To address these vulnerabilities, the framework of value-based care (VBC) or value-based healthcare (VBHC) has emerged as the definitive strategy for contemporary healthcare reform [3,4]. Developed fundamentally by Michael Porter and Elizabeth Teisberg, VBC represents a total restructuring of how healthcare is organized, delivered, measured, and reimbursed [5]. VBHC is a system where doctors and hospitals are paid based on how much they help patients, rather than how many tests or treatments they run. They are rewarded for keeping people healthy, managing chronic illnesses successfully, and preventing hospital stays. Ultimately, it focuses on delivering the best possible health results for patients at the lowest overall cost. Rather than viewing healthcare as a series of disconnected, transaction-based medical procedures, VBC positions the patient's complete care cycle as the primary unit of value creation [5,6]. This model targets the elimination of low-value clinical interventions, the minimization of medical errors, and the active optimization of long-term wellness [6,7]. By aligning the financial viability of health organizations directly with the physical recovery of populations, value-based models offer a viable mechanism to bend the healthcare cost curve while simultaneously advancing clinical excellence, institutional equity, and patient safety on a global scale [4,7]. The aim of the present study is to describe the foundations and operational mechanics of VBC as a narrative review. 

Methodology

Literature Search

This study was designed as a narrative review to synthesize current knowledge, policy frameworks, and implementation strategies regarding VBHC. A comprehensive literature search was conducted during a dedicated search period in May 2026. Two primary electronic databases were queried: PubMed (for peer-reviewed biomedical and healthcare management literature) and Google Scholar (to capture a broader spectrum of academic papers, institutional reports, and grey literature). To ensure the review incorporated high-level regulatory frameworks and health transformation goals, the electronic database search was supplemented by a manual search of grey literature. This included scanning official policy documents, strategic plans, and decrees published by relevant national and international health authorities, such as the Saudi Ministry of Health (MOH), the Center for National Health Insurance (CHI), and the Saudi Health Council.

Search Strategy

The search strategy utilized a combination of Medical Subject Headings (MeSH) terms, free-text keywords, and Boolean operators (AND, OR) to maximize search sensitivity. The search targeted literature focusing on the intersection of value-focused financing models and the Saudi Arabian healthcare ecosystem. The exact search strings applied across the databases were configured as follows:

PubMed Search String: ("value-based care"[tiab] OR "value-based health care"[tiab] OR "outcome-based finance"[tiab] OR "Value-Based Health Care"[Mesh]) AND ("Saudi Arabia"[tiab] OR "Saudi Arabia"[Mesh])

Google Scholar Search String: "value-based care" OR "value-based health care" OR "outcome-based finance" "Saudi Arabia"

No strict filters on publication date were applied during the database execution to ensure a comprehensive historical mapping of Saudi Arabia's health sector transformation, though the primary focus remained on documents aligned with the Vision 2030 timeline.

Eligibility Criteria

As a flexible narrative review, the eligibility criteria were structured to capture diverse research methodologies, expert commentaries, and authoritative policy directions. Studies explicitly discussing the concept, implementation, challenges, or financial frameworks of VBC or outcome-based financing. Publicly available documents published in English were selected.

Study Selection Process

Search results from PubMed and Google Scholar were extracted, and obvious duplicate citations were removed. The remaining records were screened by title and abstract against the inclusion criteria to filter out irrelevant clinical or strictly geographic mismatches. Parallel to the academic literature screening, targeted searches on official Saudi health portals were conducted to retrieve active policy papers and healthcare transformation blueprints. Full texts of the shortlisted articles and policy documents were retrieved and thoroughly reviewed to confirm their thematic alignment with value-based healthcare paradigms in Saudi Arabia.

Identification and Literature Synthesis

Data extraction and identification of key concepts were performed using a thematic analysis approach. A standardized narrative matrix was utilized to map out essential characteristics from each included source, including the author/organization, year of publication, specific VBC dimension (e.g., payment models, digital health enablers, patient-reported outcomes), key findings, and policy implications. Because this study is a narrative review characterizing an evolving national policy landscape, a statistical meta-analysis was neither feasible nor appropriate. Instead, the literature was synthesized using a qualitative, narrative approach.

## Review

The core philosophy: value vs. volume

To understand the core structural mechanics of VBC, it must be rigorously contrasted against the multi-payer, insurance-based, and market-driven healthcare architecture that preceded it [3,8].

The Fee-for-Service (FFS) Framework

Historically, healthcare infrastructure operated on a volume-driven, piece-rate billing structure known as fee-for-service (FFS) [3]. Under an FFS architecture, medical providers - including hospitals, specialist physicians, laboratories, and imaging clinics - are reimbursed independently for every discrete action they perform [3,4]. This means that every individual blood draw, radiological scan, pharmaceutical administration, and inpatient bed-day generates a distinct line-item invoice [4].

Because institutional revenue is linked directly to the total quantity of interventions, the system builds an inherent financial incentive for over-utilization [5,7]. Providers are economically rewarded for the density of services rather than the efficacy of the recovery process [7,8].Consequently, a hospital that suffers from high complication rates or frequent readmissions often generates more revenue under FFS than an institution that cures a patient correctly during their initial visit [8,9]. This design creates highly fragmented care, fosters systemic clinical silos, and drives up national healthcare expenditures without delivering a corresponding increase in population-level survival rates or quality of life [4,9]. While the disruption phase during the COVID-19 epidemic witnessed a huge disruption in elective services [10], the providers innovated in the operational management through applying the evidence-based strategies [11] and expanded the volume of elective services [12].

The Value-Based Framework

The value-based framework fundamentally reverses this economic equation of "more services equals to more revenue" to "better health equals to more revenue" [6]. Under VBC, reimbursement is completely decoupled from service volume [6,13]. Instead, healthcare organizations are legally, operationally, and financially incentivized to maximize patient recovery, effectively control chronic metabolic diseases, and actively eliminate redundant or unnecessary medical procedures [7,13].

Value within this paradigm is not a subjective metric of luxury or patient comfort; it is defined by a standardized mathematical formula that balances clinical outcomes against resource utilization [5]:

Value = Patient Quality & Clinical Outcomes/Total Direct and Indirect Cost of Care

In this equation, the numerator represents the specific clinical achievements that matter most to the patient - such as functional survival, speed of recovery, management of pain, and long-term avoidance of disease recurrence [5,6]. The denominator encompasses the entire financial cost required to generate those outcomes across the complete cycle of care, from initial primary diagnosis through acute hospitalization to post-acute rehabilitation [5,7]. To increase value, a healthcare system must either dramatically improve clinical outcomes at a stable cost or deliver identical clinical outcomes while significantly reducing waste and expenditure [5,13].

The four structural pillars of VBC

Successfully executing a transition from a volume-driven system to a mature VBC ecosystem requires a synchronized restructuring across four interdependent operational pillars [3,6].

Pillar 1: Preventive and Proactive Intervention

The first operational pillar focuses on moving clinical resources away from reactive acute treatment and toward proactive preventive care [3]. In acute-care-driven architectures, medical intervention peak capacity often occurs only after a patient has suffered a severe clinical deterioration - such as an acute myocardial infarction or advanced diabetic ketoacidosis [3,4]. Although the FFS model focuses on medical care, VBC systems redistribute human and financial capital to halt disease progression early [4,14]. By prioritizing regular metabolic screenings, continuous cardiovascular monitoring, timely vaccinations, and behavioral health lifestyle modifications, primary care networks intercept illnesses in their pre-clinical stages [14,15]. Managing a diabetic patient proactively in an outpatient clinic avoids acute complications, preventing expensive emergency department visits, intensive care unit admissions, and irreversible tissue damage [2,4].

Pillar 2: Integrated Care Coordination

The second pillar requires the transitioning toward integrated, multidisciplinary care teams that bridge traditional medical silos [6]. In legacy frameworks, specialists, general practitioners, acute care hospitals, and home health agencies often operate within distinct organizational structures, with limited communication or workflow integration [6,8]. VBC enforces multi-disciplinary care coordination [6]. Under this pillar, comprehensive clinical networks share a collective responsibility for the patient's entire health journey [6,16]. When a patient transitions from a tertiary surgical theatre to a regional rehabilitation center and finally back to their home environment, care coordinators ensure that all details are communicated seamlessly [16,17]. This continuous management prevents conflicting pharmaceutical prescriptions, reduces post-operative complications, eliminates the need for duplicate diagnostic testing, and shortens hospital lengths of stay [6,16].

Pillar 3: Advanced Data Interoperability and Real-World Evidence (RWE)

The technological backbone of VBC is an interoperable digital infrastructure [6,7]. To accurately measure the value equation, health systems must possess the capability to track patient health data across multiple clinical settings continuously [7]. This requires the deployment of unified electronic health records (EHRs) that utilize standardized data languages (such as fast healthcare interoperability resources (FHIR) protocols) to communicate seamlessly between different medical networks [6,18]. By gathering and analysing large pools of real-world evidence (RWE), healthcare administrators can leverage predictive machine learning algorithms to stratify patient populations by clinical risk categories [18,19]. This allows medical teams to identify highly vulnerable individuals before they decompensate, while establishing baseline metrics to benchmark individual provider performance transparently [6,18].

Pillar 4: Financial Risk-Sharing Agreements

The final pillar addresses the structural realignment of economic risk between health insurance payers and healthcare providers [2,4]. Under traditional FFS models, all financial risk is borne by the payer, who must fund every procedure ordered, regardless of its clinical utility [4]. VBC introduces contractual risk-sharing mechanisms [2,4]. These models typically function on a spectrum of risk:

One-way (upside) risk: Providers receive financial bonuses if they achieve exceptional patient outcomes below target costs, but face no financial losses if they fail to meet those goals [2].

Two-way (downside) risk: Providers actively share in the financial loss [2]. If an organization delivers poor-quality care that results in preventable complications, prolonged hospitalizations, or cost overruns, the hospital must directly reimburse the payer for a portion of those excess expenditures [2,4]. This dual structure forces clinical teams to maintain strict operational efficiency and clinical precision [4,7].

Operational taxonomy of value-based payment models

To systematically phase out the volume-driven economy, healthcare payers and government bodies utilize an operational taxonomy of distinct value-based payment models [2,4]. These structures represent varying degrees of financial risk and structural integration [4,10].

Pay-for-Performance (P4P)

Pay-for-performance serves as the foundational, least disruptive entry point into value-based reimbursement (3). In a P4P configuration, the underlying payment architecture remains anchored to the traditional FFS model; however, a portion of the provider's net reimbursement is explicitly tied to hitting specific quality, safety, and efficiency benchmarks [3,4]. Health systems track standardized performance indicators, such as the percentage of eligible patients receiving breast cancer screenings, the incidence rate of central line-associated bloodstream infections (CLABSIs), or retrospective patient experience scores [4,20]. Providers who meet or exceed these thresholds receive retrospective financial bonuses, while those who underperform face a deduction from their standard FFS schedules [3,4].

Shared Savings Models

Shared savings structures introduce a population-centric approach to healthcare economics (2). Under this model, individual physicians, specialists, and hospital groups voluntarily unite to form an accountable care organization (ACO) [2,4]. The payer calculates a historical benchmark budget representing the projected cost of caring for a defined panel of patients over a specified timeframe [4,21]. If the ACO implements care coordination strategies that keep the total realized expenditure below the baseline budget while meeting defined clinical quality standards, the financial savings are split between the insurance payer and the provider organization [2,4]. In advanced, two-way shared savings arrangements, the ACO also agrees to repay a portion of the deficit if expenditures exceed the baseline [4,21].

Bundled Payments (Episode-Based Care)

Bundled payments move away from population budgets to focus strictly on acute, well-defined clinical episodes [3,7]. Instead of issuing separate reimbursements to the surgeon, anesthesiologist, facility, and physical therapist involved in an intervention, the payer issues a single, comprehensive, fixed payment to cover the entire episode of care [3]. For example, a total knee arthroplasty bundle encompasses all preoperative testing, the acute surgical window, implant costs, inpatient hospital stays, medications, and any post-acute physical therapy required within 90 days of the operation [2,7]. If the surgical team executes the procedure efficiently and avoids readmissions, the hospital retains the financial savings [2,20]. Conversely, if clinical errors lead to wound infections or emergency readmissions, the provider network absorbs the total financial loss [7,22].

Capitation and Global Budgets

Capitation represents the highest tier of value-based purchasing, involving a total transfer of financial risk from the payer to the provider organization [1,5]. Under a capitation contract, a healthcare delivery network receives a fixed, prospective, pre-calculated fee per enrolled patient to cover all necessary medical care over a set period - typically expressed as a per-member per-month (PMPM) rate [3,23]. This payment is fixed regardless of whether an individual patient utilizes zero medical services or requires extensive multi-organ specialty interventions [3,5]. Global budgets operate similarly on a macro-institutional scale, providing an entire hospital network with a fixed annual budget to manage the total health needs of an entire region [1,23]. This structure aligns institutional survival with keeping people healthy, creating a strong incentive to optimize primary care, maximize outpatient prevention, and avoid expensive hospital bed occupancy [5].

Global implementation: The United States and Saudi Arabia

The practical implementation of VBC varies internationally, adapted to the specific socio-political, economic, and legislative environments of different countries [1,2,13].

The United States: Centers for Medicare & Medicaid Services (CMS)

In the United States, the transition toward VBC is largely spearheaded by the federal government through the Centers for Medicare & Medicaid Services (CMS) and its dedicated CMS Innovation Center [2]. Tasked with reforming a highly complex, multi-payer system, CMS has introduced a series of alternative payment models (APMs) designed to shift the country away from its historic reliance on fragmented FFS billing [2,4].

Through the ongoing evolution of the Medicare Shared Savings Program (MSSP) and the implementation of the merit-based incentive payment system (MIPS), the United States has driven a significant proportion of its healthcare providers into accountable care frameworks [4,24]. The stated strategic objective of CMS is to ensure that 100% of traditional FFS Medicare beneficiaries and the vast majority of Medicaid enrollees are managed within an accountable, value-linked care relationship by the year 2030 [2,4]. Quantitative evaluations of these programs have demonstrated documented cost reductions, particularly in post-acute care utilization, alongside marked improvements in preventive cancer screenings and long-term blood pressure control across vulnerable demographic cohorts [2,24].

The Kingdom of Saudi Arabia (KSA): Health Sector Transformation Program (HSTP)

Concurrently, the KSA is executing one of the most rapid, sweeping, and well-funded health system transformations in modern history [13,25]. Driven fundamentally by the overarching directives of Saudi Vision 2030, the MOH is systematically dismantling its legacy public health architecture through the comprehensive HSTP [13,26]. Historically, the Saudi public healthcare model relied on a centralized, supply-driven system where the government operated as both the primary funder and the direct provider of medical services, incentivizing institutional capacity and volume over specific clinical outcomes [25,27].

Under the HSTP framework, the Kingdom is enforcing a strict separation of these roles to catalyze a nationwide value-based ecosystem [13,28]. The MOH is transitioning into an independent regulatory and policymaking body, while its historic delivery assets - including hundreds of primary clinics and tertiary hospitals - are being corporatized into 20 distinct, regionally distributed, vertically integrated networks known as health clusters [13,29]. Each health cluster is structured to operate as an independent ACO, responsible for managing the total population health of a specific geographic catchment area [25,29]. Each Saudi citizen within a cluster is formally registered with a dedicated family medicine practitioner, establishing a proactive primary care foundation designed to serve as the gateway to the broader medical network [29,30].

To sustain this structural reorganization, Saudi Arabia has engineered advanced financial and regulatory bodies:

The Center for National Health Insurance (CNHI): This newly established public entity functions as the centralized national purchaser of healthcare services [13,25]. The CNHI is progressively phasing out historic line-item budgetary allocations to public hospitals [26]. Instead, it utilizes value-based purchasing methodologies to buy healthcare packages from the regional health clusters, tying public funding explicitly to the attainment of predefined health outcomes, patient satisfaction indexes, and population-level wellness metrics [13,28].

The Council of Health Insurance (CHI):** **On the private and commercial insurance front, the CHI has enacted rigorous regulatory mandates to eliminate legacy FFS billing [31]. The CHI has enforced the adoption of standardized VBHC guidelines across all private insurance contracts [31,32]. Insurance providers and private medical groups are now legally required to integrate population health management (PHM) tools, utilize standardized clinical codes, and implement risk-sharing capitation payment models designed to reward long-term health maintenance over procedural density [31].

Persistent bottlenecks and future outlook

Despite compelling theoretical advantages and strong legislative mandates, the global transition to VBC faces complex operational and cultural barriers [1,32].

Systemic Data Fragmentation and Governance Gaps

The most immediate operational hurdle is data fragmentation [6]. Many legacy healthcare systems continue to operate on proprietary, siloed technological networks that lack the capacity for bidirectional communication [6,15]. Healthcare transformation leaders note that, even in systems with heavily funded digital investments, significant gaps remain regarding raw data quality, clinical registry harmonization, and outcome metric standardization [10,30]. Without absolute data interoperability, calculating the precise clinical outcome of a patient across their complete multi-year care journey remains highly difficult, occasionally preventing accurate tracking of quality indicators [6,33]. The patient-reported outcome measures (PROMs), which are one of the foundational elements of VBC, are critically affected by data fragmentation, along with other factors [34].

Institutional and Cultural Inertia

Furthermore, the medical workforce itself frequently exhibits deep cultural inertia [29]. Clinicians, administrators, and hospital financial executives have spent decades operating within the FFS paradigm, where institutional prestige and personal income are tied directly to procedural volume, surgical frequency, and bed occupancy rates [4,8]. Research evaluating change-management within health networks emphasizes that senior professionals often view new continuous accountability care models with skepticism due to years of working in rigid, fragmented pathways [29,30]. Shifting this deeply ingrained mindset requires intense investment in clinical change management, comprehensive educational curriculum updates at medical universities, and the development of transparent performance benchmarking systems that build clinical trust [4,25], in addition to implementing strategies that incorporate the patients' opinions, preferences [35], and satisfaction [36].

Financial Micro-Costing Obstacles

From a healthcare economics perspective, determining the exact denominator of the value equation represents a significant analytical challenge [5]. Traditional hospital accounting frameworks are designed to track costs by broad department categories rather than by individual patient disease cycles [7,37]. To overcome this limitation, pioneering institutions are adopting time-driven activity-based costing (TDABC) [37,38]. TDABC requires mapping out every single step a patient takes during a clinical episode, measuring the exact amount of clinical time they spend with each provider, and accounting for the specific equipment utilized [38,39]. While highly precise, implementing TDABC at scale demands significant administrative effort and analytical expertise, creating a bottleneck for smaller community clinics and newly formed health clusters [37,39].

## Conclusions

The global transition from volume-driven FFS frameworks to patient-centric VBC structures represents an unavoidable evolution in modern health systems architecture. Driven by the unsustainable financial strain of aging populations and chronic disease epidemics, this paradigm shift systematically aligns clinical delivery with objective health outcomes. By structuring medical reimbursement around the explicit quality of recovery rather than the density of billable interventions, value-based frameworks offer a mathematically sound approach to optimizing resource efficiency while maintaining strict clinical safety and patient-reported outcomes. The present article navigates the transition of the insurance-based health system to VBHC. On a practical implementation level, the strategic realignments visible within the United States and the fast-tracked execution of the Health Sector Transformation Program in Saudi Arabia provide strong evidence for the scalability of these models. The systematic creation of corporate regional health clusters, strategic centralized purchasing via the Center for National Health Insurance, and population management rules enforced by the Council of Health Insurance showcase how a legacy system can be decentralized and integrated. When healthcare financing structures are explicitly tethered to long-term community wellness metrics and preventive data targets, the traditional operational silos separating primary clinics from tertiary hospitals naturally dissolve.

Ultimately, the long-term success of this global systemic transformation will depend heavily on overcoming the persistent bottlenecks of technical data fragmentation, micro-costing administrative workloads, and clinical change management. Achieving true digital interoperability through standardized registries remains the essential foundation needed to securely measure and track the economic value equation. As international healthcare networks continue to transition along the financial risk spectrum toward global capitation models, organizations that successfully combine data maturity with proactive primary care workflows will successfully deliver sustainable, equitable, and highly integrated care ecosystems.
